# Electrochemical Technologies to Decrease the Chemical Risk of Hospital Wastewater and Urine

**DOI:** 10.3390/molecules26226813

**Published:** 2021-11-11

**Authors:** Ángela Moratalla, Salvador Cotillas, Engracia Lacasa, Pablo Cañizares, Manuel A. Rodrigo, Cristina Sáez

**Affiliations:** 1 Department of Chemical Engineering, Faculty of Chemical Sciences and Technologies, University of Castilla-La Mancha, 13005 Ciudad Real, Spain; angela.moratalla@uclm.es (Á.M.); pablo.canizares@uclm.es (P.C.); manuel.rodrigo@uclm.es (M.A.R.); 2 Department of Chemical Engineering and Materials, Faculty of Chemical Sciences, Complutense University of Madrid, 28040 Madrid, Spain; 3 Department of Chemical Engineering, Higher Technical School of Industrial Engineering, University of Castilla-La Mancha, 02071 Albacete, Spain; engracia.lacasa@uclm.es

**Keywords:** advanced oxidation processes, pharmaceuticals, wastewater, hospital urine

## Abstract

The inefficiency of conventional biological processes to remove pharmaceutical compounds (PhCs) in wastewater is leading to their accumulation in aquatic environments. These compounds are characterized by high toxicity, high antibiotic activity and low biodegradability, and their presence is causing serious environmental risks. Because much of the PhCs consumed by humans are excreted in the urine, hospital effluents have been considered one of the main routes of entry of PhCs into the environment. In this work, a critical review of the technologies employed for the removal of PhCs in hospital wastewater was carried out. This review provides an overview of the current state of the developed technologies for decreasing the chemical risks associated with the presence of PhCs in hospital wastewater or urine in the last years, including conventional treatments (filtration, adsorption, or biological processes), advanced oxidation processes (AOPs) and electrochemical advanced oxidation processes (EAOPs).

## 1. Introduction

Pharmaceutical compounds (PhCs) play an important role in keeping worldwide human health. Most of them are synthetic polar compounds manufactured by the relevant pharmaceutical companies, although some other medical drugs are produced using biotechnology from a natural biological source (e.g., insulin). PhCs can be classified depending on their chemical nature, therapeutic actions, target anatomical regions, rate of biodegradability, bioaccumulation potential or level of hazard. The most common classification is related to their mode of action (therapeutic actions), such as analgesics, antipyretic, antibiotics, antihistamines, anti-neoplastics, β-blockers, etc. Drugs get metabolized inside the human body by the action of specific enzymes, such as cytochromes which facilitate the development of bioreactions, evolving the therapeutic actions from the active pharmaceutical ingredients (APIs). The human body may only metabolize around 60–70% of the APIs and the residual drug is excreted in urine at 55–80% followed by feces at 4–30% [1,2,3]. Subsequently, a significant amount of the excreted PhCs enter the aquatic environment in various wastewater networks.

The presence of PhCs in aquatic environments ranges from 0.1 to 100 ng/L in natural water bodies (rivers and oceans), 100–1000 ng/L in groundwaters, 1–100 ng/L in effluents from wastewater treatment plants (WWTPs), or up to 10,000 ng/L in hospital effluents [4,5,6,7]. Their persistence in aquatic ecosystems is not only a consequence of a high rate of release but of their recalcitrant nature, with it being hard to attain complete mineralization. The detected PhCs remain biologically active and cause adverse effects in nontarget organisms within aquatic life as described under the EU-Directive 93/67/EEC. Likewise, the European Union Water Framework Directive reports an updated list of priority substances every four years (2000/60/EC) where PhCs are considered as potential pollutants. Depending on their therapeutic actions, they pose various degrees of alteration threat to the natural ecological balance. Among others, antibiotics act as endocrine disruptors and are responsible for the occurrence of antibiotic-resistant microbes [8,9]. Consequently, the World Health Organization (WHO) and many other regulatory authorities have identified PhCs as emerging pollutants since they still remain unregulated or are currently undergoing a regularization process [10,11].

The discharges of human body excretions are directly flushed into municipal sewers towards the WWTPs. However, these treatment plants are designed to remove conventional pollutants from human waste, such as fats, biodegradable organic matters, nitrogen or phosphorus. Hence, the removal percentage of PhCs is lower than 10% since the biological treatment processes are not suitable to degrade complex organic molecular structures at low concentrations in water [11,12]. Among influents of WWTPs, hospital effluents are the main source of input for PhCs since they are not considered industrial effluents in most countries and, hence, regulations allow their direct discharge into the municipal sewer system without any prior treatment [13,14]. Specifically, hospital urine contains about 100 to 500 times more PhCs concentrations than domestic wastewater [15]. An efficient technological development is needed to pre-treat hospital urines as hotspots of PhCs release to ensure public health and reduce environmental risk. In this work, a review of the most recent technologies employed for the removal of PhCs in hospital wastewater (including urine matrices) is reported.

## 2. Technologies for the Removal of Pharmaceuticals in Hospital Wastewater

PhCs administered to patients admitted in the hospital are mainly excreted in urine and feces which are merged with other wastewater produced in different areas of hospital facilities, resulting in hospital wastewater (HWW) [16,17]. Specifically, HWW involves the effluents generated from sanitary activities (clinical treatments), toilets (urine, feces…), kitchen, laundry, or garden among others, which contain large amounts of chemicals, organic matter (including microorganisms: bacteria, virus and fungi) and inorganic ions [16]. Table 1 shows the typical composition of these effluents reported in the literature [18,19,20,21,22,23,24].

Chloride is the ion in the highest concentration, whereas urea is the main organic compound found in these effluents. This can be due to the use of large amounts of chlorine-based disinfectants in hospital facilities for cleaning activities and, the human urine from patients and the health staff which contains large concentrations of urea. Furthermore, HWW has a range of concentrations of 0.0001 to 100 mg dm^−3^ of pharmaceuticals in their composition, which mainly include antibiotics (up to 100 mg dm^−3^), analgesics and anti-inflammatories (up to 40 mg dm^−3^), beta blockers and hypertensives (up to 20 mg dm^−3^), antidepressants (up to 0.008 mg dm^−3^) and anticonvulsants (up to 0.005 mg dm^−3^). These compounds are not degraded in conventional WWTPs and they are released to the environment [25]. For this reason, the development and application of efficient technologies for decreasing the risks associated to the presence of PhCs in sanitary effluents is critical from an engineering and environmental viewpoint.

HWW also contains high levels of microbiological contaminants, such as bacteria (*Escherichia coli*, *Enterococci*, fecal coliforms, total coliforms…), viruses (Enteroviruses, astroviruses, norovirus, hepatitis A…), fungi, etc. Thus, the development of these technologies could favor the elimination of not only PhCs but also microbiological content [26]. These microorganisms can be eliminated under milder conditions than PhCs by chlorination, ultraviolet, ozone, Fenton process, photocatalysis, etc. [27,28,29], or by in situ generation of oxidizing species (advanced electrochemical oxidation processes) [30,31,32].

**Figure 1:**
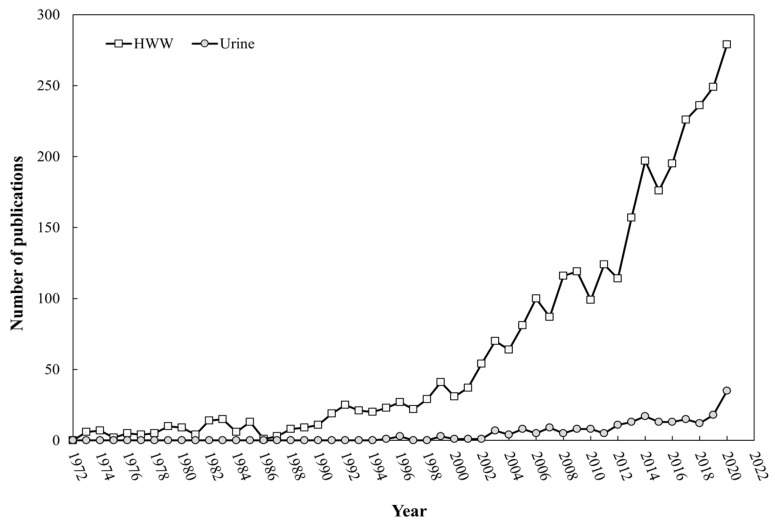
Publications related to the removal of PhCs in HWW and only hospital urine from 1970 to 2020.

The lack of legislation regulating the levels of PhCs in HWW promotes a rapid spread and accumulation of these compounds in the environment [17]. This also involves a health problem since favors the occurrence of ARB. Nonetheless, concern in the scientific community related to the development of highly efficient technologies for removing PhCs in hospital wastewater has increased considerably in recent years. Figure 1 summarizes the number of publications reported on the degradation of PhCs in hospital effluents (including hospital urine) and only urine reported from the early 70s.

As can be observed, the number of publications has increased over the years, being more remarkable from the 2000s. Specifically, the manuscripts per year are lower than 50 up to 2001 and then, significantly increase until reach more than 250 publications in 2020. This reveals the growing interest from the scientific community in the treatment of HWW for the removal of PhCs as a pre-treatment before discharge to conventional WWTPs since the concentration of these pollutants is expected to be higher and, hence, easier to detect and treat. Even so, only 6.81% of the total publications summarized in Figure 1 referred to the removal of PhCs in urine.

### 2.1. Conventional Processes

Biological and physical-chemical processes have been tested for the removal of PhCs in HWW due to their low cost and ease of operation. Table 2 summarizes the most relevant conventional technologies for this purpose reported in the literature until 2021.

**Figure 2:**
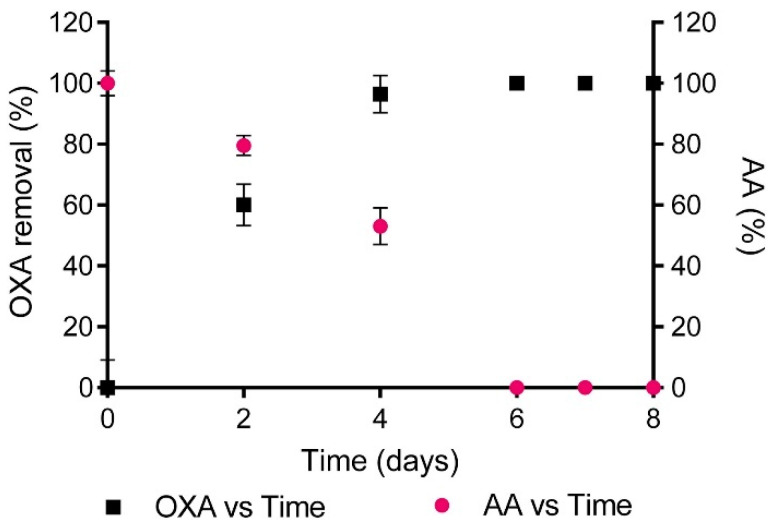
Evolution of oxacillin concentration and antibacterial activity (AA) as a function of the operating time during the biological degradation process by *Leptosphaerulina* sp. Reprinted with permission from ref. [20]. Copyright 2018 Elsevier.

The use of a microbial consortium with *Pseudomonas aeruginosa* (*P. aeruginosa*), *Citrobacter freundii*, *Klebsiella pneumoniae* and *Escherichia coli* were tested for the removal of 40 mg dm^−3^ dicloxacillin in HWW, finding that it was possible to completely remove the antibiotic in less than 4 h [33]. Likewise, the biological degradation of dicloxacillin was also studied with *P. aeruginosa* but, in this case, an operating time of more than 50 h was required to achieve the complete antibiotic removal. These results reveal that the antibiotic degradation efficiency can be significantly improved using a microbial consortium under the operating conditions tested. Copete-Petuz et al. [20] evaluated a Colombian native fungus (*Leptosphaerulina* sp.) for the removal of 16 mg dm^−3^ oxacillin. Conical flasks were inoculated and incubated at 28 °C with agitation (160 rpm) for 8 days and, the antibiotic was completely degraded in 6 days (Figure 2).

Despite biological processes being effective and low-cost for the complete removal of PhCs in HWW, the operating times required to achieve a significant degradation of these compounds can be very high. Hence, other chemical processes have been evaluated for this purpose with the aim of obtaining high removal efficiencies and low operating times. The use of carbon-based materials has been reported for the adsorption of PhCs contained in HWW [19,34,35]. Lima et al. [22] studied the elimination of acetaminophen (40–80 mg dm^−3^) from HWW using activated carbon derived from Brazil nutshells (BN) with ZnCl_2_. Removal percentages higher than 95% were achieved in 30 min using different ratios ZnCl_2_/BN, regardless of the initial concentration of the pollutant. Furthermore, the removal of antibiotic amoxicillin (30–60 mg dm^−3^) using activated carbon with *Bertholletia excelsa* capsules (CPP) was evaluated by Lima et al. The adsorbents were prepared with a ratio of 1:1 ZnCl_2_:CPP, and the mixture was pyrolyzed at 600 and 700 °C, reaching removal percentages higher than 97% in 30 min in all cases [36]. On the other hand, magnetic adsorbents from olive kernels (MA-OK) were employed for the removal of high concentrations of amoxicillin (200–400 mg dm^−3^) in HWW by Jafari et al. [37]. They concluded that the use of adsorbent doses of 0.5 g dm^−3^ at pH 6 led to removal percentages within the range 89–98% in 90 min.

Another interesting process for the elimination of PhCs in HWW is electrochemical coagulation [18,38]. This technology consists of the generation of coagulant species from the electrodissolution of a sacrificial anode that allows for the removal of pollutants by different physical-chemical mechanisms, such as charge neutralization or sweep flocculation [43]. The removal of 154 µg dm^−3^ ciprofloxacin in HWW using electrocoagulation with aluminium electrodes was reported by Ahmadzadeh et al. [39]. Total antibiotic removal was attained in 20 min when applying 12.5 mA cm^−2^ at pH 7.78. Malakootian et al. [40] evaluated the application of electrocoagulation with aluminium electrodes and persulfate for the removal of 3.5 mg dm^−3^ ciprofloxacin in HWW, reaching an elimination percentage higher than 81% in 40 min. During this process, persulfate can be activated electrochemically, favoring antibiotic degradation. Hence, ciprofloxacin is not only removed by physical separation promoted by electrocoagulation, but also can be chemically attacked by activated persulfate. Likewise, the treatment of HWW by the combination of electrocoagulation with other physical processes has been reported in the literature. Ahmadzadeh et al. [41] studied the removal of 60 mg dm^−3^ cefazolin in HWW by electrocoagulation with aluminium electrodes combined with adsorption using chitosan. The antibiotic was eliminated in 23 min, applying a current density of 15.5 mA cm^−2^ and a chitosan concentration of 0.7 g dm^−3^ at pH 7.8.

Membrane technologies have also been tested for the removal of PhCs in HWW. The application of nanofiltration to the treatment of urine polluted with anticancer drugs was studied by Cristóvão et al. [42]. Two different membranes were evaluated (Desal 5 DK and NF270) for the elimination of paclitaxel, etoposide, cyclophosphamide and ifosfamide with an initial concentration of 0.5 mg dm^−3^. The Desal 5 DK membrane has a molecular weight cut-off between 150 and 300 Da, whereas NF270 has a molecular weight cut-off of 300 Da. Removal percentages higher than 95% were attained for paclitaxel and etoposide, regardless the membrane used. However, Desal 5 DK membrane led to removal percentages higher than 96% for cyclophosphamide and ifosfamide whereas the use of the NF270 membrane achieved values higher than 80% for these compounds. This reveals that the Desal 5 DK membrane is more suitable for the removal of anticancer drugs from urine.

### 2.2. Advanced Oxidation Processes (AOPs)

Biological and physical-chemical technologies allow to remove PhCs from HWW, however, in many cases, the pollutants are not destroyed but only separated by adsorbents or flocs without altering their structure. For this reason, the application of Advanced Oxidation Processes (AOPs) to treat HWW has become a promising alternative to degrade PhCs. These technologies involve all processes that promote the generation of large amounts of highly reactive species for pollutants degradation. AOPs can be divided into two major groups: homogeneous and heterogeneous, which, in turn, can be classified into two different groups, depending on the energy requirements [44]. Table 3 summarizes the most relevant AOPs reported in the literature until 2021 for the degradation of PhCs in HWW.

Ozone (E^0^: 2.08 V) is a powerful oxidant that can be decomposed to form the hydroxyl radical (E^0^: 2.80 V), a more oxidizing and non-selective species capable of destroying organic pollutants contained in water bodies. This process can be carried out in alkaline media to promote the rapid decomposition of ozone (non-catalytic ozonation) or using solid catalysts (catalytic ozonation) [58,59]. Agudelo et al. [45] evaluated the removal of 6 mg dm^−3^ meropenem in HWW by catalytic ozonation using powder activated carbon-Portland cement as a catalyst. They applied an ozone flow rate of 37.5 mg O_3_ min^−1^ and reached the total removal of antibiotics in less than 12 min.

Another oxidant species that can be activated to produce large amounts of hydroxyl radicals is hydrogen peroxide (E_0_: 1.78 V). The use of iron-based catalysts for this purpose is well known as Fenton reaction (Equation (1)) [60,61].

H_2_O_2_ + Fe^2+^ → ·OH + Fe^3+^ + OH^−^ (1)

**Figure 3:**
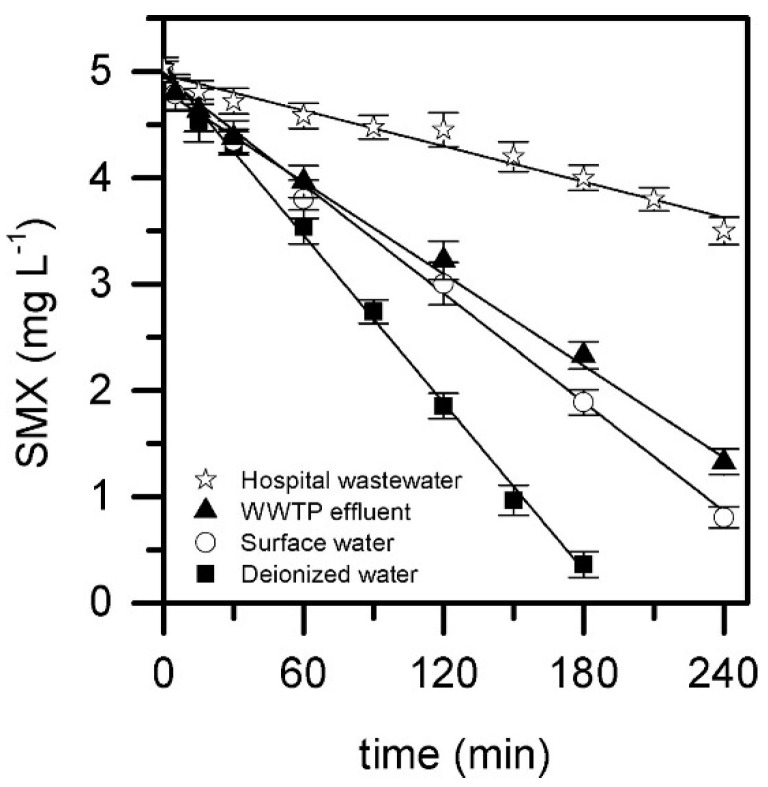
Evolution of SMX upon CWPO with magnetite in different real aqueous matrices ([SMX]_0_ = 5 mg L^−1^; [H_2_O_2_]_0_ = 25 mg L^−1^; [magnetite]_0_ = 1 g L^−1^; pH_0_ = 5; T = 25 °C). Experimental (symbols) and model fit (solid lines). Reprinted with permission from ref. [47]. Copyright 2018 Elsevier.

The degradation of antibiotic sulfamethoxazole by Fenton process was reported by Wu et al. [46] who studied the activation of hydrogen peroxide by a Fe-Mn binary oxide (FMBO). The initial amount of hydrogen peroxide employed was 6 mM with a catalyst concentration of 2 g dm^−3^. The complete degradation of 0.1 mg dm^−3^ sulfamethoxazole was attained in 10 min and a percentage removal higher than 90% was achieved at the same time (10 min) during the treatment of HWW polluted with 1.6 mg dm^−3^ sulfamethoxazole. Muñoz et al. [47] evaluated the removal of 5 mg dm^−3^ sulfamethoxazole in HWW by Fenton process at pH 5 using 25 mg dm^−3^ H_2_O_2_ and 1 g dm^−3^ magnetite as catalyst (heterogeneous Fenton). They reached a removal percentage of around 30% in 240 min (Figure 3).

The Fenton process can be enhanced by the irradiation of UV light (photo-Fenton) since it promotes the massive production of hydroxyl radicals from the photoactivation of both hydrogen peroxide and catalyst, depending on the wavelength applied [62,63,64]. Papoutsakis et al. [48] studied the treatment of urine polluted with iohexol (600–6000 mg dm^−3^) by photo-Fenton under simulated solar light. A constant UVA intensity of 30 W m^−2^ was applied to polluted urine containing 400 mg dm^−3^ H_2_O_2_ and 20 mg dm^−3^ Fe^2+^ at pH 3. Results showed that it was possible to attain removal percentages higher than 95% in 120 min during the treatment of diluted urine (600 mg dm^−3^ iohexol) and values around 50% in 360 min when treating urine directly (6000 mg dm^−3^). On the other hand, the treatment of HWW polluted with 50 µg dm^−3^ anastrozole by solar photo-Fenton was reported by Sanabria et al. [49]. They used 25 mg dm^−3^ H_2_O_2_ and a constant catalyst concentration of 10 mg dm^−3^ at pH 5, achieving removal percentages around 50% in 120 min.

Several studies have shown that the combination of the Solar photo-Fenton process with tertiary processes (such as adsorption) can improve the removal of persistent pharmaceutical compounds [65,66,67]. In this context, Della-Flora et al. [50] investigated the degradation of Flutamide (500 μg L^−1^) and its transformation products (TPs) from HWW by Solar photo-Fenton combined with adsorption with activated carbon. Solar photo-Fenton was applied using three Fe^2+^ additions approach (5 mg L^−1^ of Fe^2+^ each, with an initial H_2_O_2_ concentration of 150 mg L^−1^) achieving 58% degradation in 120 min. For the adsorption process, 14 mg of avocado seed activated carbon was used and a contact time of 40 min, obtaining Flutamide and TPs degradation rates of over 97%.

**Figure 4:**
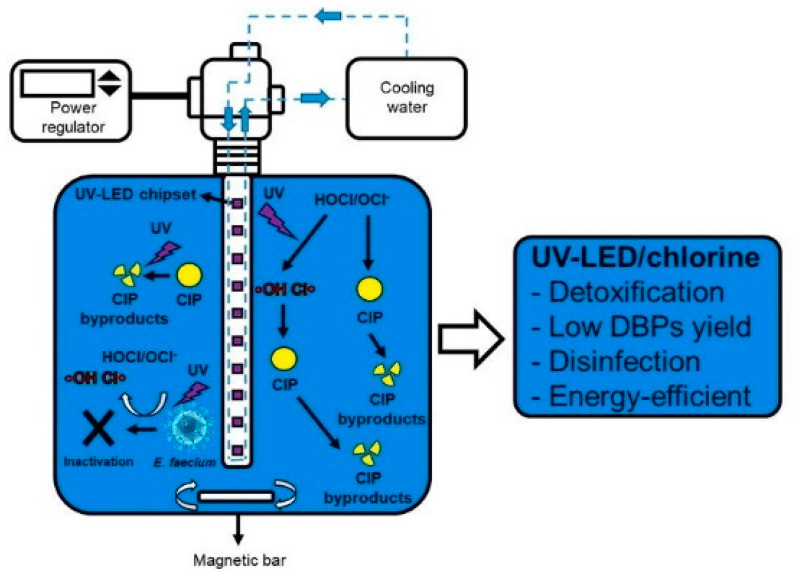
Schematic diagram of UV-LED reactor. Reprinted with permission from ref. [52]. Copyright 2020 Elservier.

The irradiation of UV light has also been tested in the photoactivation of hydrogen peroxide (UVC/H_2_O_2_) or chlorine (UVC/Cl_2_) for the treatment of HWW. In these cases, a wavelength around 254 nm (UVC) is required to ensure the decomposition of hydrogen peroxide and chlorine to free radicals [68,69]. Jaén-Gil et al. [51] reported the degradation of metoprolol (2 µg dm^−3^) and metoprolol acid (2 µg dm^−3^) in HWW by UVC/H_2_O_2_ using 25 mg dm^−3^ H_2_O_2_ and a UVC_254_ nm lamp of 15 W. Removal percentages higher than 70% were achieved in 10 min, being the degradation of metoprolol acid faster than that of metoprolol (88.7 vs. 71.6%). Kim et al. [52] evaluated the treatment of ciprofloxacin polluted HWW by UVC/Cl_2_ at pH 7 in a UV-LED reactor (Figure 4). Chlorine doses of 15 mg dm^−3^ were added to the effluents under UV-LED irradiation (275 nm), reaching the complete removal of 10 mg dm^−3^ ciprofloxacin in 60 min.

Another AOP that employs the irradiation of UV light to produce reactive oxidizing species (ROS) is photocatalysis. During this process, a semiconductor material absorbs UV light for moving an electron from the valence gap to the conduction band. This generates a positive hole in the valence band that can oxidize H_2_O or OH^−^, favoring the production of ROS [70]. The most common photocatalyst used for the removal of organic pollutants in wastewater is titanium dioxide (TiO_2_) [71]. Chinnaiyan et al. [53] reported the removal of metformin (10 mg dm^−3^) and amoxicillin (10 mg dm^−3^) in HWW by photocatalysis using TiO_2_ as photocatalyst (563 mg dm^−3^) and a UV lamp of 125 W (365 nm). The process was carried out at pH 7.6 and the results showed that it was possible to attain removal percentages higher than 90% for both PhCs in 150 min. Furthermore, the elimination of lorsatan from urine by photocatalysis with TiO_2_ was studied by Guateque-Londoño et al. [54]. They used 0.5 g dm^−3^ TiO_2_ and UVA light irradiation (75 W) at pH 6.1 for the degradation of 43.38 µmol dm^−3^ lorsatan, reaching removal percentages around 35% in 20 min. Other photocatalysts based on ZnO have also been tested for the removal of organic pollutants. Gharaghani et al. [55] evaluated the elimination of 3 mg dm^−3^ ciprofloxacin in HWW using ZnO nanoparticles at pH 11. The antibiotic was almost completely removed (90.25%) in 90 min under the operating conditions tested.

On the other hand, AOPs based on persulfate have been studied for the treatment of HWW and urine. This oxidant species can be photoactivated by the irradiation of UV light, favoring the production of free sulfate radicals (Equation (2)) which can attack organic pollutants contained in the effluents.

S_2_O_8_^2−^ + h*v* → 2 SO_4_^−^ (2)

**Figure 5:**
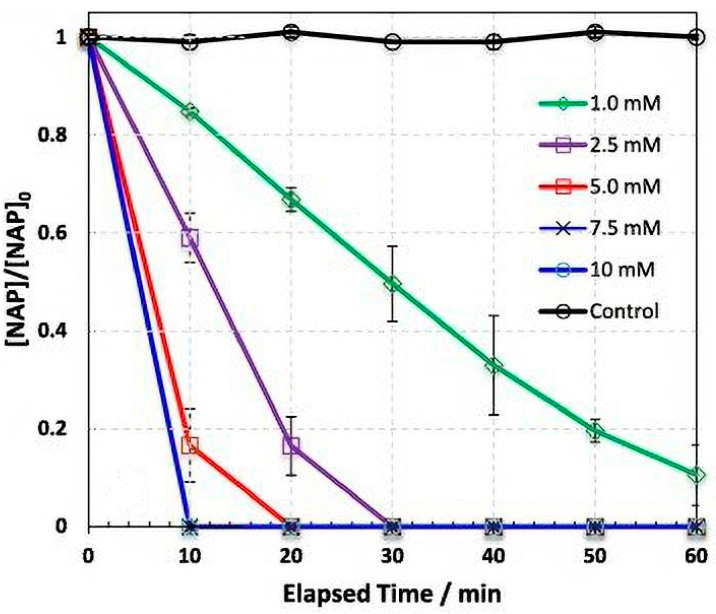
Evolution of naproxen concentration as function of the elapsed time during the treatment of hospital effluents. [NAP]0 = 50 μÌM, pH 7.50, T = 70 °C. Reprinted with permission from ref. [56]. Copyright 2015 Elsevier.

Guateque-Londoño et al. [54] evaluated the degradation of 43.38 µmol dm^−3^ lorsatan in urine using 500 µmol dm^−3^ S_2_O_8_^2−^ and UVC light (60 W) at pH 6.1. Removal percentages around 35% were achieved in 20 min. Persulfate can also be activated by heating to produce sulfate radicals [72,73]. The elimination of 50 µM naproxen in HWW by thermally activated persulfate was reported by Ghauch et al. [56]. An initial concentration of 10 mM S_2_O_8_^2−^ was added to the effluent at pH 7.5 and, the temperature was increased up to 70 °C. The complete removal of PhC was attained in 10 min (Figure 5).

The integration of biological processes with AOPs can also increase the efficiency of the treatments. Jaén-Gil et al. [57] evaluated the combination of UV/H_2_O_2_ with a biological process (with activated sludge) for the removal of metropolol (2 μg L^−1^) and metropolol acid (2 μg L^−1^) from HWW. They proposed two different configurations: biological process + AOP and AOP + biological process. The removal rates of metropolol and metropolol acid were 85.7% and 98.5%, respectively, during the sequence biological process + AOP. However, the degradation efficiencies increased when AOP + biological process was carried out. Specifically, removal percentages of 85.6% and 99.5% were achieved for metropolol and metropolol acid, respectively. Furthermore, the intermediate compounds were removed up to 85%. This reveals that the sequence AOP + biological process improves the removal of metoprolol and metoprolol acid from HWW.

### 2.3. Electrochemical Advanced Oxidation Processes (EAOPs)

AOPs based on electrochemical technology have been recently applied to the degradation of PhCs in hospital wastewater [74,75,76,77]. These processes are commonly called Electrochemical Advanced Oxidation Processes (EAOPs) and, promote the generation of large amounts of highly reactive species from the in-situ oxidation and reduction reactions induced in the effluents without the addition of chemicals for the removal of organics [78]. The selection of appropriate electrode materials and reactor design are critical for developing highly efficient EAOPs [79,80]. Likewise, the current density is the most influential operating parameter for the development and scale-up of EAOPs. Table 4 summarizes the most relevant EAOPs reported in the literature until 2021 for the degradation of PhCs in HWW.

**Figure 6:**
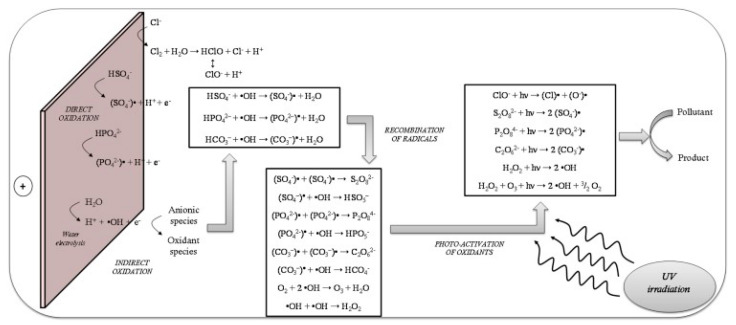
Mechanisms expected for the photo-electrolytic reclamation of secondarily treated wastewater. Reprinted with permission from ref [98]. Copyright 2016 Elsevier.

Electrochemical oxidation is the most widely used EAOP for the removal of organic pollutants in water matrices [81,82,83]. Specifically, this process consists of the abatement of organics in an electrolytic cell by different mechanisms: (i) direct electron transfer to the anode and (ii) indirect or mediated oxidation by highly reactive species formed from water discharge at the anode surface [84]. Figure 6 shows the main mechanisms of the process related to oxidants production and activation [98].

The anode materials used for the development of this process can be classified as active and non-active anodes. The first ones favor the chemisorption of in situ electrogenerated free radicals on the anode surface whereas non-active anodes promote the physisorption of these species [99]. Materials based on Pt, IrO_2_ and RuO_2_ are examples of active anodes, and diamond-based coatings, SnO_2,_ or PbO_2_ are considered as non-active anodes [100]. The application of electrooxidation to the treatment of HWW polluted with cephalexin was studied by Serna-Galvis et al. [85] using a Ti/IrO_2_ anode. The antibiotic (40 µM) removal rate was approximately 60% after 30 min, applying a current density of 5 mA cm^−2^ at pH 6.5. The presence of significant amounts of chloride ions in the effluent promoted the electrochemical production of free chlorine by anodic oxidation, which improved the degradation of the antibiotic by an indirect oxidation mechanism. The same experimental set-up and electrodes materials (Ti/IrO_2_ anode and zirconium spiral cathode) were used by [86]. In this case, they studied the simultaneous degradation of diclofenac (40 µM) and naproxen (40 µM) in urine at 5 mA cm^−2^ and pH 6.0. Results showed elimination rates of 30% for diclofenac and 20% for naproxen in 30 min of electrolysis.

**Figure 7:**
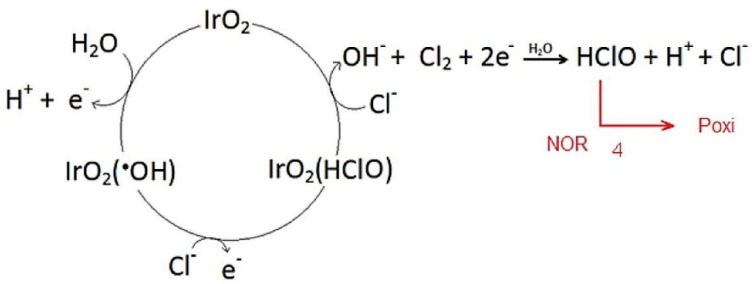
Main electrochemical degradation pathway of norfloxacin in presence of chloride ions. Reprinted with permission from ref. [87]. Copyright 2017 Elsevier.

A Ti/IrO_2_ anode (Figure 7) was also tested by Jojoa-sierra et al. [87] for the removal of 125 μM norfloxacin in urine applying 6.53 mA cm^−2^. An antibiotic removal percentage of around 65% was attained at 180 min since the oxidation of urea competes with the degradation of norfloxacin during the electrolysis of urine.

Sordello et al. [88] evaluated the feasibility of the electrooxidation process for the removal of Cefazolin (100 μM) from urine using a platinum sheet anode and a glassy carbon cathode. The range of current densities was 0.5–150.0 mA cm^−2^. They concluded that Cefazolin can be degraded at current densities from 0.5, 5.0, 50.0 and 150.0 mA cm^−2^ at approximate electrolysis times of 500, 160, 40 and 10 min, respectively. Zwiener et al. [89] used a platinum net as anode and used a reticulated nickel foam electrode as a cathode to remove 0.1 mM of iomeprol (iodinated contrast media) in urine. The voltage applied during electrooxidation was 1V. Complete removal of iomeprol was achieved after 120 min of electrolysis.

**Figure 8:**
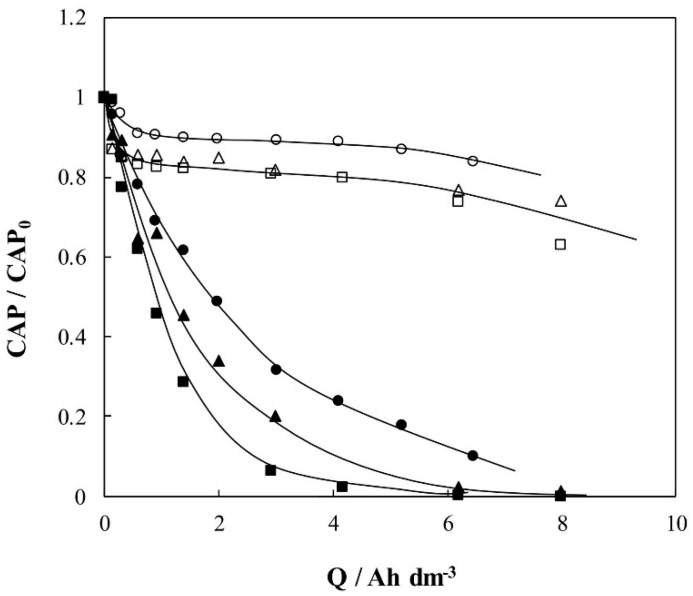
Evolution of chloramphenicol as a function of the applied electric charge during the electrochemical oxidation of 100 mg dm^−3^ CAP in urine media. Current density: (■, □) 1.25 mA cm^−2^; (▲, ∆) 2.5 mA cm^−2^; (●, ○) 5 mA cm^−2^. Anodic material: (black symbols) BDD; (white symbols) MMO. Reprinted with permission from ref [90]. Copyright 2020 Elservier.

On the other hand, the degradation of a mixture of PhCs (analgesics, antibiotics, antihypertensive, caffeine) in HWW with concentrations ranging from 0.16 µg L^−1^ to 93 µg L^−1^ by electrochemical oxidation was reported by Ouarda et al. [21]. Boron doped diamond was used as an anode, Ti as cathode and the applied current densities between both electrodes were within the range 4.42–35.4 mA cm^−2^. Results showed that pharmaceutical abatement rates were greater than 50% after 120 min of electrolysis when applying 35.4 mA cm^−2^. More recently, Herraiz-Carboné et al. [90] compared the use of active and non-active anodes for the removal of 100 mg dm^−3^ chloramphenicol in urine. They concluded that it was possible to attain a complete antibiotic removal when working with BDD anodes for all the current densities tested (1.25–5 mA cm^−2^) whereas the use of anodes based on mixed metal oxides (MMO) led to removal percentages of around 25% under the same operating conditions (Figure 8). Free and combined chlorine species were generated during the treatment of urine from the oxidation of chlorides which contributed to the degradation of antibiotics with both anodes. Nonetheless, the use of BDD anodes also promoted the electrochemical generation of peroxocompounds, such as persulfate or peroxodiphosphate from the oxidation of other ions contained in urine, favoring antibiotic removal.

To reduce the costs and energy consumption of the electrochemical processes for the removal of PhCs, some authors have evaluated the combination of electrooxidation with biological processes. Ouarda et al. [91] reported the treatment of HWW contaminated with carbamazepine (10 µg L^−1^), ibuprofen (10 µg L^−1^), estradiol (10 µg L^−1^) and venlafaxine (0.2 µg L^−1^) using a membrane bioreactor technology combined with the electrooxidation process. They compared the removal efficiencies of the different PhCs using two treatment configurations: electrooxidation process as pre-treatment and post-treatment. Results showed that the most effective combination was the application of electrooxidation as a post-treatment (MBR-EO), achieving removal rates of over 97% for all PhCs tested after 40 min, applying a current intensity of 0.5 A with Nb/BDD as electrodes.

**Figure 9:**
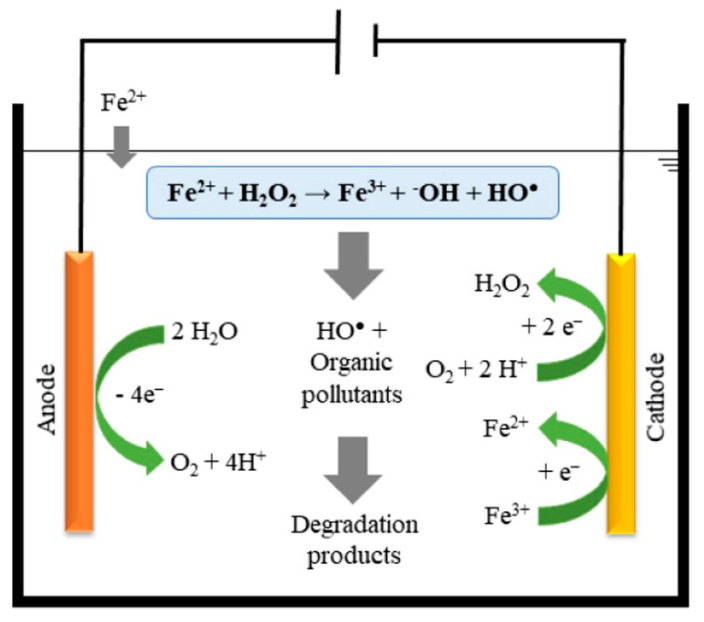
Mechanisms expected in the electro-Fenton process. Reprinted with permission from ref [102]. Copyright 2021 Elsevier.

Another environmentally friendly EAOP applied to the removal of PhCs in water bodies is electro-Fenton [101]. This process starts with the in situ electrogeneration of hydrogen peroxide (H_2_O_2_) in the solution by the reduction of oxygen at the cathode according to Equation (3). Then, hydroxyl radicals are homogeneously produced in the bulk from the reaction between electrogenerated H_2_O_2_ and ferrous ion (catalyst) externally added at low pH values (Fenton reaction) (Equation (1)). Figure 9 shows the main mechanisms involved in the electro-Fenton process.

One of the advantages of the electro-Fenton over classical Fenton process (where the reagents are added chemically) is that the catalyst (Fe^2+^) can be continuously electrogenerated through Equation (4), promoting the catalytic cycle required by the Fenton system. Furthermore, the use of non-active anodes, such as diamond-based coatings during the electro-Fenton process generates an additional source of ·OH which are heterogeneously formed over the anode surface through water oxidation (Equation (5)).

O_2_ (g) + 2H^+^ + 2 e^−^ → H_2_O_2_ (3)

Fe^3+^ + e^−^ → Fe^2+^ (4)

H_2_O → ·OH + H^+^ + e^−^ (5)

**Figure 10:**
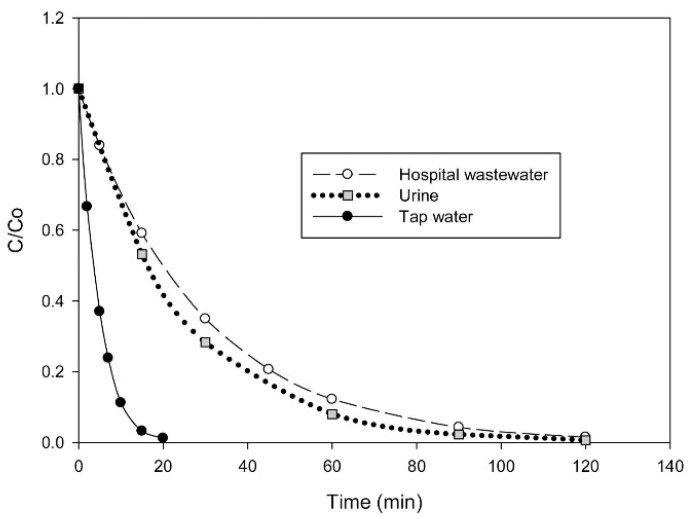
Degradation of piroxicam in different matrices. Experimental conditions: [Piroxicam] = 0.08 mM; [Na_2_SO_4_] = 0.05 M; [Fe^2+^] = 0.10 mM; I = 100 mA (4.17 mA cm^−2^); V = 0.25 L; pH = 3.0 and room temperature. Reprinted with permission from ref. [92]. Copyright 2019 Elservier.

Feng et al. [92] evaluated the removal of 0.08 mM piroxicam in HWW and urine by electro-Fenton at pH 3, using BDD and 3D-carbon-felt as anode and cathode, respectively. The catalyst concentration employed was 0.1 mM Fe^2+^ and the current density applied was 4.17 mA cm^−2^. Complete elimination was attained after 120 min in both effluents, being slower than the results obtained during the treatment of tap water (Figure 10). This can be related to the occurrence of oxidative competitive reactions between the PhC and other organics, such as urea or acetate contained in HWW and urine. On the other hand, the treatment of HWW polluted with 1.35 mg dm^−3^ acetaminophen by electro-Fenton was reported by Ahmadzadeh et al. [93]. They used two iron plate electrodes at 8 mA cm^−2^, 122.5 μL dm^−3^ H_2_O_2_ and pH 2.75. The ferrous iron required for carrying out the Fenton reaction was in situ electrogenerated by the electrodissolution of the anode. Results showed that it was possible to attain the complete elimination of acetaminophen after 10 min.

One of the main disadvantages of the electro-Fenton process is the low solubility of oxygen in water at atmospheric pressure, which significantly influences the production of hydrogen peroxide at the cathode. To overcome this limitation, Moratalla et al. [94] recently reported the use of a pressurized electrochemical reactor equipped with a jet aerator for the removal of meropenem in urine, demonstrating that the electrochemical generation of hydrogen peroxide can be significantly improved by applying high pressures. Specifically, they evaluated the influence of pressure (gauge pressure range of 0 to 3 bar) on the elimination of 50 mg dm^−3^ meropenem in urine by the heterogeneous electro-Fenton process, using a 3D-MMO-IrO_2_Ta_2_O_5_ mesh anode and a modified 3D-titanium mesh with CB/PTFE cathode at 5 mA cm^−2^, pH 3 and 10.8 g goethite (heterogeneous catalyst). Results confirmed that the meropenem degradation rate increased with the gauge pressure. The antibiotic removal percentages attained were 80.60, 89.03, 91.60 and 94.64% at gauge pressures of 0, 1, 2 and 3 bar, respectively, when passing 0.8 Ah dm^−3^ at 5 mA cm^−2^.

EAOPs can be enhanced by the irradiation of UV light to promote the photoactivation of electrogenerated oxidants, favoring the production of free radicals that significantly contribute to the degradation of organic pollutants [103]. Specifically, free chlorine (Equation (6)) and sulfate (Equation (2)) radicals can be generated by the photoactivation of electrogenerated hypochlorite and persulphate with UVC light, respectively [104].

ClO^−^ + h*v* → Cl^·^ + O^−^ (6)

Gonzaga et al. [95] compared the elimination of 50 mg dm^−3^ penicillin G in urine matrixes by electrolysis and photoelectrolysis with active anodes (MMO-Ti/RuO_2_IrO_2_). They used a UVC lamp of 9 W, and a current density applied of 30 mA cm^−2^. Results showed a marked synergistic effect on the degradation of the antibiotic when coupling UVC light to electrolysis, reaching a total removal of the pollutant in 8 h. The degradation of penicillin G was also studied by Gonzaga et al. [96], comparing the electro-Fenton and photoelectron-Fenton processes under acidic conditions (pH 3). Two different anodes were used (BDD and MMO-Ti/Ru_0.5_Ir_0.5_O_2_) and a modified carbon-felt was employed as the cathode. The catalyst concentration was 0.5 mM Fe^2+^ and the current intensity was 120 mA. They reported that the influence of the anode material is less relevant, although MMO led to faster penicillin G removal than BDD anode. The antibiotic degradation was enhanced during the photoelectron-Fenton process since the photoactivation of hydrogen peroxide by UVC light irradiation can also take place (Equation (7)), increasing the production of free hydroxyl radicals in the effluent.

H_2_O_2_ + h*v* → 2 ·OH (7)

**Figure 11:**
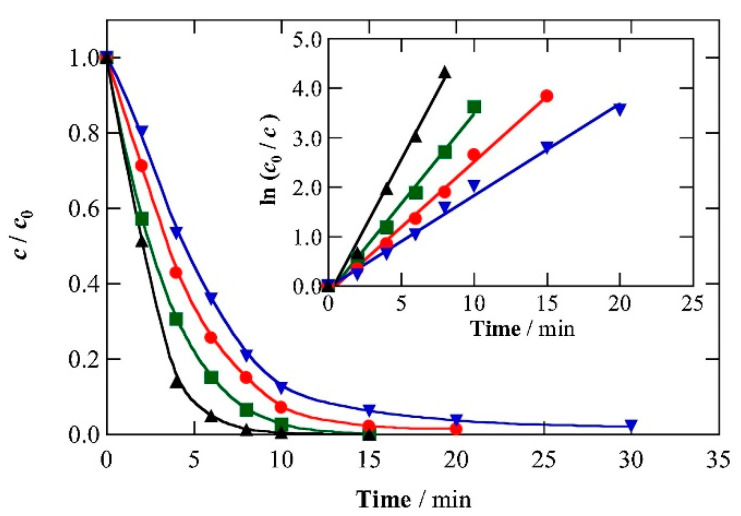
Influence of the aqueous matrix on the normalized captopril concentration decay during the SPEF treatment of 2.5 L of 0.230 mM drug solutions with 0.50 mM Fe^2+^ at pH 3.0 and 35 °C using a solar pre-pilot flow plant with a Pt/air-diffusion cell at *j* = 50 mA cm^−2^ and liquid flow rate of 180 L h^−1^. Matrix: (▲) Urban wastewater, (■) urine 1 (13.9 mM urea + 0.073 mM uric acid + 0.367 mM creatinine), (●) urine 2 (27.8 mM urea + 0.146 mM uric acid + 0.734 mM creatinine) and (▼) urine 3 (55.6 mM urea + 0.292 mM uric acid + 1.47 mM creatinine). The inset panel shows the kinetic analysis of the above concentration decays assuming a pseudo-first-order reaction. Reprinted with permission from ref. [80]. Copyright 2020 Elsevier.

Finally, Dos Santos et al. [80] evaluated the removal of captopril (0.23 mM) from urine in three different synthetic urine matrices (Urine 1, Urine 2 and Urine 3) by Solar photo Electro-Fenton. In this case, the photolytic action of sunlight (UVA light) is used for enhancing the performance of the electro-Fenton process. The experiments were carried out in a solar pre-pilot flow plant, where the anode was a Pt plate, and the cathode was a carbon-PTFE air diffusion electrode. The initial amount of Fe^2+^ was 0.5 mM at pH 3 and 50 mA cm^−2^. Each synthetic urine matrix presents other organic compounds in different concentrations: creatinine, urea and uric acid, where Urine 1 is the most dilute and Urine 3 is the most concentrated. Although these organic compounds slow down the process, captopril abatement was achieved at 15, 20 and 30 min during the treatment of urine 1, 2 and 3, respectively (Figure 11).

Another important point in EAOPs processes is the design of the cell/electrochemical reactors with the aim of improving PhCs removal efficiencies and reducing operational costs [105]. In this design, it is important to consider the configuration of the reactor (conventional stirred-tank cell, flow-by reactor, or flow-through reactor) as well as the geometry of the electrode (plane, mesh, foam). Gonzaga et al. [97] compared two reactor configurations (a conventional stirred-tank cell and a microfluidic flow-through reactor) in the removal of three antibiotics (penicillin G, meropenem and chloramphenicol; 50 mg dm^−3^ each) in urine by electrooxidation and photo-electrooxidation. In the microfluidic flow-through reactor, the anode support material used was a porous titanium foam (3D-electrode) and in the conventional stirred-tank cell used a titanium plate (2D-electrode). In both cases, the composition of the electrode was MMO-Ti/RuO_2_IrO_2_ and the current density was 30 mA cm^−2^. Results show that when using the microfluidic flow-through reactor, the reaction rate is much faster (from 2–4 times) than when using the conventional stirred tank. For example, in the photo-electrooxidation process, the conventional cell is able to remove up to 82% of each of the antibiotics at 6.4 Ah dm^−3^. However, the microfluidic cell achieves complete removal of all three antibiotics for the same applied charge. Another important difference is that the electrical consumption to oxidize the antibiotics in urine is about three times lower in the microfluidic flow-through. This improvement can be attributed to the larger active area of the anode (3D-foam), the improved mass transport coefficient and the decreased ohmic resistance in the microfluidic flow-through.

## 3. Conclusions

The occurrence of PhCs in water bodies has increased over the years, with hospital wastewater as the major source of these pollutants. For this reason, to preserve the aquatic environment, it is necessary to know the type and levels of PhCs contained in hospital effluents. Conventional biological processes have been tested to biodegrade antibiotics using bacteria, such as *Pseudomonas aeruginosa*, microbial consortium, or fungi, such as the Colombian native fungus (*Leptosphaerulina* sp.). In addition, different activated carbons prepared with Caesalpinia ferrea, Brazil nutshells with ZnCl_2_, *Bertholletia excelsa* or kenaf, as well as magnetic adsorbents from olive kernels (MA-OK) have been used in the adsorption process. Another conventional treatment, such as electrochemically assisted coagulation has been combined with the adsorption process using chitosan to improve the degradation efficiencies in HWW.

AOPs have also been tested for the removal of PhCs in hospital wastewater and urine. These technologies promote the generation of highly reactive species for the degradation of organic pollutants. Fenton-based processes have been employed for the removal of PhCs in hospital effluents using Fe, Fe-Mn binary oxide, or magnetite as catalysts. The coupling of UV light irradiation to these technologies (photo-Fenton) was checked for the removal of PhCs, in order to improve the removal efficiencies. Likewise, photocatalytic processes using TiO_2_ as a photo-catalyst have also been tested for the removal of PhCs in this type of effluents. On the other hand, persulfate-based AOPs have been studied for the treatment of hospital wastewater. The enhancement of these processes can be favored by the irradiation of UV light to form free sulfate radicals by the photo-activation of persulfate.

Within AOPs, EAOPs are considered as a new alternative for the degradation of PhCs in hospital wastewater where oxidizing species are in-situ generated from the oxidation and reduction reactions in the system. These processes can also be enhanced by the coupling of irradiation technologies (UVA, UVC and solar irradiation). Electrochemical oxidation has been extensively studied for the elimination of PhCs in hospital wastewater using different electrodes (active and non-active anodes). Likewise, the electro-Fenton process (using different anodic and cathodic materials) has proven to be a promising technology for the removal of PhCs in hospital effluents.

## Figures and Tables

**Table 1:** Composition of HWW.

Parameters	Units	Range	Compound	Units	Range
HCO_3_^−^	mg dm^−3^	0–85	Saccharose	mg dm^−3^	0–30
CO_3_^2−^		0–6	Glucose		0–30
Cl^−^		50–2000	COD	mg O_2_ dm^−3^	300–420
SO_4_^2−^		4–70	BOD_5_		187–304
Ca^2+^		2–20	pH	-	7.0–7.5
K^+^		3–75	Antibiotics		0.0001–100
Mg^2+^		2–4	Analgesics and anti-inflammatories	mg dm^−3^	0.00013–40
Na^+^		25–1200	Betablocker		10–20
S^2−^		0–15	Hypertensive		10–20
PO_4_^3-^		5–30	Antidepressant		0.00387–0.008
NO_3_^−^		0–10	Anticonvulsants		0.0006–0.005
NH^4+^		10–70	*Enterococci*	UCF mL^−1^	10^3^–10^6^
Urea		10–1300	*Escherichia coli*		10^3^–10^6^
Humic acid	mg dm^−3^	0–10	Fecal coliforms	CFU mL^−1^	10^3^–10^4^
Citric acid		0–10	Total coliforms		10^5^–10^7^

**Table 2:** Conventional processes for the removal of PhCs in HWW.

Effluent	Technology	Operation Parameters	Target Drug	Concentration	% Elimination	Ref.
HWW	Electrocoagulation	Aluminium electrodes (61 cm^2^), monopole configuration. 1000 mA	Dexamethasone	100 μg L^−^^1^	~30 (45 min)	[18]
HWW	Adsorption	Porous activated carbons prepared with Caesalpinia ferrea.	Captopril	25 mg L^−1^	CFAC.0.5/89.63 (60 min)	[19]
					CFAC.1.0/95.96 (60 min)	
		CFAC 0.5 (ratio of 0.5:1.0 of ZnCl_2_/CF at 600 °C)			CFAC.1.5/97.67 (60 min)	
		CFAC 1.0. (ratio of 1.0:1.0 of ZnCl_2_/CF at 600 °C)		50 mg L^−1^	CFAC.0.5/86.08 (60 min)	
					CFAC.1.0/92.07 (60 min)	
		CFAC 1.5. (ratio of 1.5:1.0 of ZnCl_2_/CF at 600 °C)			CFAC.1.5/94.22 (60 min)	
HWW	Biological	*Leptosphaerulina sp.* (a Colombian native fungus). Conical flaks are inoculated and incubated at 28 °C and 160 rpm for 8 days.	Oxacillin	16 mg L^−1^	100 (6 days)	[20]
HWW	Adsorption	Activated carbons derived from Brazil nutshells: BNS1.0 (ratio of 1.0:1.0 of ZnCl_2_/BN at 600 °C) BNS1.5 (ratio of 1.5:1.0 of ZnCl_2_/BN at 600 °C)	Acetaminophen	40 mg L^−1^	BNS1.0/98.29 (30 min) BNS1./98.83 (30 min)	[22]
				80 mg L^−1^	BNS1.0/96.38 (30 min) BNS1.5/97.04 (30 min)	
HWW	Biodegradation (Biological)	*Pseudomonas aeruginosa* (1.5 × 10^8^ CFU mL^−1^)	Dicloxacillin	40 mg L^−1^	100 (52 h)	[33]
		Microbial consortium (*Pseudomonas aeruginosa, Citrobacter freundii, Klebsiella pneumoniae, and Escherichia coli*) (1.5 × 10^8^ CFU mL^−1^)			100 (3.75 h)	
HWW	Adsorption	Activated carbon filters with different concentrations of kenaf: K-36-500/36% K-60-500/60% K-85-500/85%	Paracetamol	120 mg L^−^^1^	K-36-500/~42 (1000 min) K-60-500/~83 (1000 min) K-85-500/~68 (1000 min)	[34]
HWW	Adsorption	Sawdust adsorbent modified. Adsorbent dose 3.6 g L^−^^1^ and pH 8.3	Tetracycline	~0.25 mg L^−1^	~100 (53 min)	[35]
HWW	Adsorption	Activated carbons with *Bertholletia excelsa* capsules: CCP.600 (ratio of 1.0:1.0 of ZnCl_2_/CCP at 600 °C) CCP.700 (ratio of 1.0:1.0 of ZnCl_2_/CCP at 700 °C)	Amoxicillin	30 mg L^−1^	CCP.600/98.01 (30 min) CCP.700/98.60 (30 min)	[36]
				60 mg L^−1^	CCP.600/97.28 (30 min) CCP.700/97.76 (30 min)	
HWW	Adsorption	Magnetic adsorbent was prepared from Olive kernel (MA-OK). Adsorbent dose = 0.5 g L^−^^1^, pH = 6	Amoxicillin	200 mg L^−^^1^	95.31 (90 min)	[37]
				300 mg L^−^^1^	89.81 (90 min)	
				400 mg L^−^^1^	97.90 (90 min)	
HWW	Electrocoagulation	Three aluminium plates anodes and three iron plates cathodes. V = 30 V, pH = 7	Cefazolin	0.0423 mg L^−^^1^	94 (30 min)	[38]
HWW	Electrocoagulation	Two aluminium plate electrodes at 12.5 mA cm^−2^; pH = 7.78	Ciprofloxacin	154 μg L^−^^1^	100 (20 min)	[39]
HWW	Electrocoagulation-persulfate	Two aluminium anodes and two aluminium cathodes at 2.75 mA cm^−2^; pH = 7. PS concentration of 0.84 mM	Ciprofloxacin	3.5 mg L^−^^1^	81 (40 min)	[40]
HWW	Electrocoagulation-adsorption	Aluminium electrodes at pH 7.8, 15.5 mA cm^−2^, 0.7 g L^−^^1^ chitosan	Cefazolin	60 mg L^−^^1^	100 (23 min)	[41]
Urine	Nanofiltration	Stainless steel dead-end stirred cell with an area of 54 cm^2^: Desal 5 DK membrane (150–300 Da) NF270 membrane (300 Da)	Paclitaxel Etoposide Cyclophosphamide Ifosfamide	0.5 mg L^−^^1^	Desal 5 DK >95/>95/96.6/96.3	[42]
					NF270 >95/>95/81.1/82.5	

**Table 3:** AOPS for the removal of PhCs in HWW.

Effluent	Technology	Operation Parameters	Target Drug	Concentration	% Elimination	Ref.
HWW	Catalytic Ozonation	37.5 mg O_3_/min	Meropenem	6 mg L^−1^	100 (11.7 min)	[45]
HWW	H_2_O_2_/Fe-Mn binary oxide	[H_2_O_2_]_0_ = 6.0 mM, 2.0 g L^−1^ of Fe-Mn binary oxide	Sulfamethoxazole	0.1 mg L^−1^	100 (10 min)	[46]
				1.6 mg L^−1^	92.8 (10 min)	
HWW	H_2_O_2_/magnetite	[H_2_O_2_] = 25 ppm; [Magnetite] = 1 g L^−1^; pH_0_ = 5; T = 25 °C.	Sulfamethoxazole	5 mg L^−1^	~30 (240 min)	[47]
Urine	Photo-Fenton	Simulated solar light at constant UVA intensity of 30 W m^−2^. 20 ppm Fe^2+^, pH = 3. [H_2_O_2_]_0_ = 400 mg L^−1^ (replenished when it dropped below 100 mg L^−1^). Two types of urine: diluted 1:10 and undiluted.	Iohexol	600 mg L^−1^	Diluted urine ~95 (120 min)	[48]
				6000 mg L^−1^	Undiluted urine ~48 (360 min)	
HWW	Solar Photo-Fenton	[H_2_O_2_]_0_ = 25 mg L^−1^, multiple addition of iron = 10 mg L^−1^ and pH = 5.0.	Anastrozole	50 μg L^−1^	~50 (120 min)	[49]
HWW	Solar Photo-Fenton and adsorption	Solar Photo Fenton process: three Fe^2+^ additions (5 mg dm^−3^ Fe^2+^ each and 150 mg dm^−3^) Adsorption: 14 mg of avocado seed activated carbon	Flutamide and transformation products	500 μg L^−1^	Solar Photo-Fenton: 58 (120 min) Adsorption: >97 (40 min)	[50]
HWW	UV/H_2_O_2_	Photo-oxidation process. UV_254_ lamp (15 W), [H_2_O_2_]_0_ = 25 mg L^−1^	Metoprolol	2.0 μg L^−1^	71.6 (10 min)	[51]
			Metoprolol acid	2.0 μg L^−1^	88.7 (10 min)	
HWW	UV (275 nm)/ Chlorination	Glass reactor with magnetic stirrer. UV-LED of 275 nm. [Free available chlorine] = 15 mg L^−1^, pH = 7	Ciprofloxacin	10 mg L^−1^	100 (60 min)	[52]
HWW	TiO_2_-photocatalysis	Laboratory-scale photoreactor. UV lamp (365 nm) = 125 W. pH 7.6, TiO_2_ dosage is 563 mg L^−1^	Metformin	10 mg L^−1^	98 (150 min)	[53]
			Amoxicillin	10 mg L^−1^	90 (150 min)	
Urine	TiO_2_-photocatalysis	[TiO_2_]: 0.5 g L^−1^, pH: 6.1, UVA light: 75 W	Losartan	43.38 μmol L^−^	~35 (20 min)	[54]
Urine	UV/Persulfate	[PS] = 500 μmol L^−1^, pH = 6.1, UVC light: 60 W.	Losartan	43.38 μmol L^−1^	~35 (20 min)	[54]
HWW	Nano-photocatalysis	ZnO concentration on the plat: 0.6 g L^−1^. pH = 11, reaction time 90 min.	Ciprofloxacin	3 mg L^−1^	90.25 (90 min)	[55]
HWW	Thermally activated persulfate	Sodium persulfate = 10 mM, phosphate buffer = 50 μM. 20 mL, pH = 7.5, T = 70 °C.	Naproxen	50 μM	~100 (10 min)	[56]
HWW	UV/H_2_O_2_ and biological process	Photo-oxidation process: Immersion-type photo-reactor. UV lamp (15 W), [H_2_O_2_]: 15 mg L^−1^ with a reaction time of 10 min. Bioreactor with activated sludge were operated as a batch with reaction time of 24 h	Metoprolol Metropolol acid	2.0 μg L^−1^ 2.0 μg L^−1^	Bioreactor-UV/H_2_O_2_ 85.7 98.5 UV/H_2_O_2_-Bioreactor 85.6 99.5	[57]

**Table 4:** EAOPS for the removal of PhCs in HWW.

Effluent	Technology	Operation Parameters	Target Drug	Concentration	% Elimination	Ref.
HWW	Electrooxidation	Two circular mesh anodes (Nb/BDD)/cathodes (Ti) at 35.4 mA cm^−2^. Flowrate: 1 L min^−1^	Caffeine	93 µg L^−1^	>50 (120 min)	[21]
			Dihydrocabamazenine	4.9 µg L^−1^		
			Desvenlafaxine	8 µg L^−1^		
			Sulfamethoxazole	3 µg L^−1^		
			Venlafaxine	3.87 µg L^−1^		
			2-Hydroxy Ibuprofen	69 µg L^−1^		
			Carbamazepine	0.62 µg L^−1^		
			4-Hydroxy Diclofenac	0.13 µg L^−1^		
			Diclofenac	0.16 µg L^−1^		
			Ibuprofen	20 µg L^−1^		
			Clarithromycin	0.06 µg L^−1^		
HWW	Electrooxidation	Flow-through electrochemical cell. BDD electrodes layer at 0.9 and 3.1 A and 50 °C	Iopromide 17-alpha-ethinylestradiol Sulfamethoma-zole Diclofenac	0.5 or 10 mg L^−1^	0.5 mg L^−1^-0.9 A: ~32/95/99/87 (180 min) 0.5 mg L^−1^-3.1 A: ~78/100/100/100 (180 min) 10 mg L^−1^-3.1 A: ~100/100/100/100 (540 min)	[74]
Urine	Electrooxidation	BDD anodes with boron content of 100, 200, 1300, 2500 and 8000 ppm and stainless steel (cathode) at 30.00 mA cm^−2^	Penicillin G	50 mg L^−1^	BDD100/98.03 at 6.4 Ah dm^−3^ BDD200/100.00 at 6.4 Ah dm^−3^ BDD1300/94.50 at 6.4 Ah dm^−3^ BDD2500/89.90 at 6.4 Ah dm^−3^ BDD8000/94.29 at 6.4 Ah dm^−3^	[75]
Urine	Electrooxidation	Single compartment electrochemical cell. BDD anode at 10 and 100 mA cm^−2^ MMO anode at 10 and 100 mA cm^−2^	Penicillin G	100 mg L^−1^	BDD: 100.00 (10 mA cm^−2^; 2.60 Ah dm^−3^)/100.00 (100 mA cm^−2^; 1.54 Ah dm^−3^) MMO:100.00 (10 mA cm^−2^; 12.30 Ah dm^−3^) /100.00 (100 mA cm^−2^; 5.61 Ah dm^−3^)	[76]
Urine	Electrooxidation	Pair of platinum-based iridium oxide composite electrodes at 1 A. The urine was diluted 2-fold, 4-fold and 8-fold.	Methotrexate	880.2 μM	2-fold/98.66 (4 h) 4-fold/99.98 (4 h) 8-fold/100.00 (4 h)	[77]
Urine	Electrooxidation	Anodic oxidation-H_2_O_2_. Three types of anodes. BDD, Pt and IrO_2_. Cathode: carbon-PTFE air diffusion electrode, pH = 3 at 33.3 mA cm^−2^	Captopril	0.23 mM	BDD anode:100.00 (60 min) Pt anode: 100.00 (60 min) IrO_2_ anode: 87.00 (60 min)	[80]
Urine	Solar Photo Electro-Fenton	A solar planar pre-pilot flow plant. Anode: Pt plate. Cathode: carbon-PTFE air diffusion electrode. Flow rate: 180 L h^−1^ and 0.5 mM Fe^2+^ at 50 mA cm^−2^ and pH 3 and 35 °C Three synthetic urine solutions Urine 1: 13.9 mM urea + 0.073 mM uric acid + 0.367 mM creatinine Urine 2: 27.8 mM urea + 0.146 mM uric acid + 0.734 mM creatinine Urine 3: 55.6 mM urea + 0.292 mM uric acid + 1.470 mM creatinine	Captopril	0.23 mM	Urine 1: 100 (15 min) Urine 2: 100 (20 min) Urine 3: 100 (30 min)	[80]
Urine	Electrooxidation	One-compartment filter-press flow cell. Flow rate: 460 mL min^−1^. Ti/Ru_0.3_Ti_0.7_O_2_ DSA^®^ at 10,20,30 and 40 mA cm^−2^	Tetracycline	200 mg L^−1^	10 mA cm^−2^: ~52.00 (3 h) 20 mA cm^−2^: ~83.00 (3 h) 30 mA cm^−2^: ~99.00 (3 h) 40 mA cm^−2^: ~100.00 (3 h)	[81]
Urine	Electrooxidation	MMO-Ti/RuO_2_-IrO_2_ anode and zirconium spiral (cathode) at 4.0 mA cm^−2^	Cephalexin	86.0 μM	~100.00 (2 h or 0.43 Ah dm^−3^)	[82]
Urine	Electrooxidation	BDD with 500 ppm of boron (Diacell cell) at 20, 50 and 100 mA cm^−2^. Flow rate: 6.67 mL s^−1^. Urine in methanol.	17-β Estradiol	10 mg L^−1^	20 mA cm^−2^: 100~7 Ah dm^−3^ 50 mA cm^−2^:100~13 Ah dm^−3^ 100 mA cm^−2^:100~15 Ah dm^−3^	[83]
Urine	Electrooxidation	Single compartment electrochemical cell. BDD anode with boron content of 500 ppm at 100 and 1000 A m^−2^	Ibuprofen Cloxacillin	10 mg L^−1^ 1 mg L^−1^	100 A m^−2^: Ibuprofen/100~32 Ah dm^−3^; Cloxacillin/100 18 Ah dm^−3^ 1000 A m^−2^: Ibuprofen/100~28 Ah dm^−3^; Cloxacillin/100~13 Ah dm^−3^	[84]
HWW	Electrooxidation	Ti/IrO_2_ rectangular (anode) and zirconium spiral (cathode). pH = 6.5 at 5 mA cm^−2^	Cephalexin	40 µM	~60 (30 min)	[85]
Urine	Electrooxidation	Undivided cell equipped with a Ti/IrO_2_ anode and a zirconium spiral cathode. pH = 6.0 and 5 mA cm^−2^	Naproxen Diclofenac	40 µM 40 µM	20 (60 min) 30 (60 min)	[86]
Urine	Electrooxidation	MMO-Ti/IrO_2_ anode and Titanium cathode at 6.53 mA cm^−2^	Norfloxacin	125.0 μM	~65 (180 min)	[87]
Urine	Electrooxidation	Undivided cell. Pt sheet was used as anode and a glassy carbon was used as cathode. Current density range: 0.5–150.0 mA cm^−2^	Cefazolin	100.0 μM	0.5 mA cm^−2^: ~100 (500 min) 5.0 mA cm^−2^: ~100 (160 min) 50.0 mA cm^−2^: ~100 (40 min) 150.0 mA cm^−2^: ~100 (10 min)	[88]
Urine	Electrooxidation	A platinum net was used as anode and reticulated nickel foam electrode was used as cathode and. V: 1 V	Iomeprol	0.1 mM	100 (120 min)	[89]
Urine	Electrooxidation	Single compartment electrochemical cell. BDD anode and stainless steel (cathode) at 1.25, 2.5 and 5 mA cm^−2^. MMO-RuO_2_ anode and stainless steel (cathode) at 1.25, 2.5 and 5 mA cm^−2^.	Chloramphenicol	100 mg L^−1^	BDD at 1.25 mA cm^−2^/100 (8 Ah dm^−3^) BDD at 2.5 mA cm^−2^/100 (8 Ah dm^−3^) BDD at 5 mA cm^−2^ /~90 (6.46 Ah dm^−3^) MMO at 1.25 mA cm^−2^/36.86 (8 Ah dm^−3^) MMO at 2.5 mA cm^−2^/25.88 (8 Ah dm^−3^) MMO at 5 mA cm^−2^/16.26 (6.46 Ah dm^−3^)	[90]
HWW	MBR-Electrooxidation	Submerged membrane bioreactor (MBR) in continuous mode. Electrooxidation reactor in discontinuous mode. Nb/BDD anode at 0.5 A.	Carbamazepine Ibuprofen Estradiol Venlafaxine	10 µg L^−1^ 10 µg L^−1^ 10 µg L^−1^ 0.2 µg L^−1^	MBR-EO ~97 (40 min)	[91]
HWW/urine	Electro-Fenton	BDD anode, 3D-Carbon-felt (cathode), 0.1 mM Fe^2+^ pH: 3 at 4.17 mA cm^−2^	Piroxicam	25.6 mg L^−1^	100 (120 min)	[92]
HWW	Electro-Fenton	Two iron plate electrodes. 2.75 pH solution, 122.5 μL L^−1^ H_2_O_2_ and 8 mA cm^−2^	Acetaminophen	1.35 mg L^−1^	100 (10 min)	[93]
Urine	Electro-Fenton	Microfluidic Flow-Through reactor. Pressurized system. 3D-MMO-IrO_2_Ta_2_O_5_ anode and modified 3D-titanium mesh with CB/PTFE cathode, pH 3, 5 mA cm^−2^, and 10.8 g goethite (heterogeneous catalyst). Gauge pressure range: 0, 1, 2 and 3 bar	Meropenem	50 mg L^−1^	0 bar: 80.60 (0.8 Ah dm^−3^) 1 bar: 89.03 (0.8 Ah dm^−3^) 2 bar: 91.60 (0.8 Ah dm^−3^) 3 bar: 94.64 (0.8 Ah dm^−3^)	[94]
Urine	Electrooxidation and photo-electro oxidation	Microwave-made MMO-Ti/RuO_2_IrO_2_ anode and stainless steel (cathode). BDD anode with a boron content of 200 ppm and stainless steel (cathode). Current density: 30 mA cm^−2^. UVC lamp 9W in photo-electrooxidation.	Penicillin G	50 mg L^−1^	EO-MMO: ~94.0 (8 h) EO-BDD: ~89.0 (8 h) PhEO-MMO: ~100.0 (8 h) PhEO-BDD: ~98.0 (8 h)	[95]
Urine	Electro-Fenton or photo Electro-Fenton	Two different anode: 200 ppm BDD and a MMO- Ti/Ru_0.5_Ir_0.5_O_2_. Cathode: modified carbon felt. 120 mA. 0.5 mM of Fe^2+^, pH 3 and a 9W UVC lamp for the PhEF tests	Penicillin G	50 mg L^−1^	EF-MMO: 99.0 (8 h) EF-BDD: 98.4 (8 h) PhEF-MMO: 100.0 (8 h) PhEF-BDD: 99.6 (8 h)	[96]
Urine	Electrooxidation and photo-electro oxidation	Two experimental configurations: Conventional stirred-tank Anode: 2D-MMO-Ti/RuO_2_IrO_2_ plate Cathode: stainless steel Microfluidic Flow-Through Anode: 3D-MMO-Ti/RuO_2_IrO_2_ foam Cathode: stainless steel Current density: 30 mA cm^−2^. UVC lamp 9 W in photo-electrooxidation.	Penicillin G Meropenem Chloramphenicol	50 mg L^−1^ 50 mg L^−1^ 50 mg L^−1^	Conventional stirred-tank: EO: >70% (6.4 Ah dm^−3^) PhEO: 82% (6.4 Ah dm^−3^) Microfluidic Flow-Through EO > 70% (6.4 Ah dm^−3^) PhEO: 100% (6.4 Ah dm^−3^)	[97]

## Data Availability

Not applicable.
